# Long Tandem Arrays of *Cassandra* Retroelements and Their Role in Genome Dynamics in Plants

**DOI:** 10.3390/ijms21082931

**Published:** 2020-04-22

**Authors:** Ruslan Kalendar, Olga Raskina, Alexander Belyayev, Alan H. Schulman

**Affiliations:** 1 Department of Agricultural Sciences, University of Helsinki, P.O. Box 27 (Latokartanonkaari 5), FI-00014 Helsinki, Finland; 2 RSE “National Center for Biotechnology”, Korgalzhyn Highway 13/5, Nur-Sultan 010000, Kazakhstan; 3 Institute of Evolution, University of Haifa, Mount Carmel, Haifa 31905, Israel; raskina@research.haifa.ac.il; 4 Laboratory of Molecular Cytogenetics and Karyology, Institute of Botany of the ASCR, Zámek 1, CZ-252 43 Průhonice, Czech Republic; alexander.belyayev@ibot.cas.cz; 5 Natural Resources Institute Finland (Luke), Latokartanonkaari 9, FI-00790 Helsinki, Finland; 6 Institute of Biotechnology and Viikki Plant Science Centre, University of Helsinki, P.O. Box 65, FI-00014 Helsinki, Finland

**Keywords:** retrotransposon, *Cassandra* TRIM, 5S RNA gene, long tandem array, ectopic recombination, genome evolution

## Abstract

Retrotransposable elements are widely distributed and diverse in eukaryotes. Their copy number increases through reverse-transcription-mediated propagation, while they can be lost through recombinational processes, generating genomic rearrangements. We previously identified extensive structurally uniform retrotransposon groups in which no member contains the *gag*, *pol*, or *env* internal domains. Because of the lack of protein-coding capacity, these groups are non-autonomous in replication, even if transcriptionally active. The *Cassandra* element belongs to the non-autonomous group called terminal-repeat retrotransposons in miniature (TRIM). It carries 5S RNA sequences with conserved RNA polymerase (pol) III promoters and terminators in its long terminal repeats (LTRs). Here, we identified multiple extended tandem arrays of *Cassandra* retrotransposons within different plant species, including ferns. At least 12 copies of repeated LTRs (as the tandem unit) and internal domain (as a spacer), giving a pattern that resembles the cellular 5S rRNA genes, were identified. A cytogenetic analysis revealed the specific chromosomal pattern of the *Cassandra* retrotransposon with prominent clustering at and around 5S rDNA loci. The secondary structure of the *Cassandra* retroelement RNA is predicted to form super-loops, in which the two LTRs are complementary to each other and can initiate local recombination, leading to the tandem arrays of *Cassandra* elements. The array structures are conserved for *Cassandra* retroelements of different species. We speculate that recombination events similar to those of 5S rRNA genes may explain the wide variation in *Cassandra* copy number. Likewise, the organization of 5S rRNA gene sequences is very variable in flowering plants; part of what is taken for 5S gene copy variation may be variation in *Cassandra* number. The role of the *Cassandra* 5S sequences remains to be established.

## 1. Introduction

Repetitive sequences are extensively distributed throughout the genomes of many organisms [1,2,3,4,5,6]. A repetitive sequence refers to highly similar DNA fragments that are present in multiple copies in the genome. Tandem direct repeats represent arrays of DNA fragments immediately adjacent to each other in head-to-tail orientation [7,8,9,10,11,12]. The eukaryotic ribosomal RNA gene (rDNA) families typically display tandem-repeat structures and are present in long arrays. The rDNA genes are generally clustered in long tandem repeats at one or several loci in most eukaryotic genomes. A common feature of these repeats is their copy number instability [1,7,13,14,15]. Various mechanisms have been proposed for the fluctuation of repeat copy number [16]. Variation in copy number is a common characteristic of rDNAs [17,18,19] and has been reported in many organisms including yeast, *Drosophila*, *Arabidopsis*, and in humans [20]. Human 5S rDNA consists of 2.2 kb repeating elements that include the 5S rRNA gene. These repeats are organized in tandem arrays and their copy number in normal individuals varies from 35 to 175 copies per haploid genome. In contrast, the human genome project estimated only 17 repeats, probably due to the known difficulties in the assembly of sequenced tandem arrays, similar to tandemly repeated *Alu* elements (which belong to an order of non-autonomous retroelements termed short interspersed elements or SINEs; [2]) and 5S rRNA genes [21].

Retrotransposons constitute a major component of dispersed repetitive DNA in all eukaryotic genomes. One of the major orders of these elements, the long terminal repeat retrotransposons (RLX; [2]), replicates by cycles of transcription, reverse transcription, and integration of daughter copies back into the genome [22,23,24]. Retrotransposons co-opt the host cell machinery to transcribe their genomic DNA copies into RNA: this RNA serves both as the messenger RNA (mRNA) that is used to make the encoded proteins and as the genomic RNA (gRNA) that is reverse-transcribed into complementary DNA (cDNA). The cDNA copies can be integrated into new chromosomal sites (“copy and paste” transposition) in the nucleus. Two conserved groups of LTR retrotransposons have been described that lack protein-coding capacity. Members of one group, the LARDs (LArge Retrotransposon Derivatives), contain a core domain but encode no protein products [25]. In contrast, the members of the second group, TRIMs (Terminal-Repeat retrotransposons In Miniature), have short LTRs and lack internal domains almost entirely [26,27,28,29]. Because they do not encode their own proteins, it is assumed that both the TRIMs and LARDs depend on trans-complementation by the products of autonomous retrotransposons for mobility.

*Cassandra* retrotransposons belong to the TRIM group and universally carry conserved sequences similar to the 5S rRNA gene and its associated RNA polymerase III promoters in their long terminal repeats [30,31]. *Cassandra* elements are widespread throughout the vascular plants, ferns, and both monocotyledonous and dicotyledonous angiosperms [30]. The organization and distribution of the *Cassandra* elements in plant genomes is still unexplored. Therefore, we aimed to detect and evaluate genome organization of this element as a contribution to understanding its role in genome size variation.

Here, we report that *Cassandra* elements with their 5S components are arranged into tandem arrays in various plant species, as is typical of the 5S rRNA genes encoding the ribosomal 5S components. When the *Cassandra* retroelements are transcribed from long tandem arrays, their RNA forms a special secondary structure. The secondary structure of the *Cassandra* transcript forms super-loops, in which both the LTR strands form the stems, with both strands being complementary to each other in a highly stable manner. The internal domain of the *Cassandra* retroelement forms the loop in the secondary structure, which may contribute to the recombination process leading to the formation of long tandem arrays.

## 2. Results

### 2.1. In Silico Identification of the Cassandra Retrotransposon and Long Tandem Arrays in Plant Genomes

**Figure 1:**
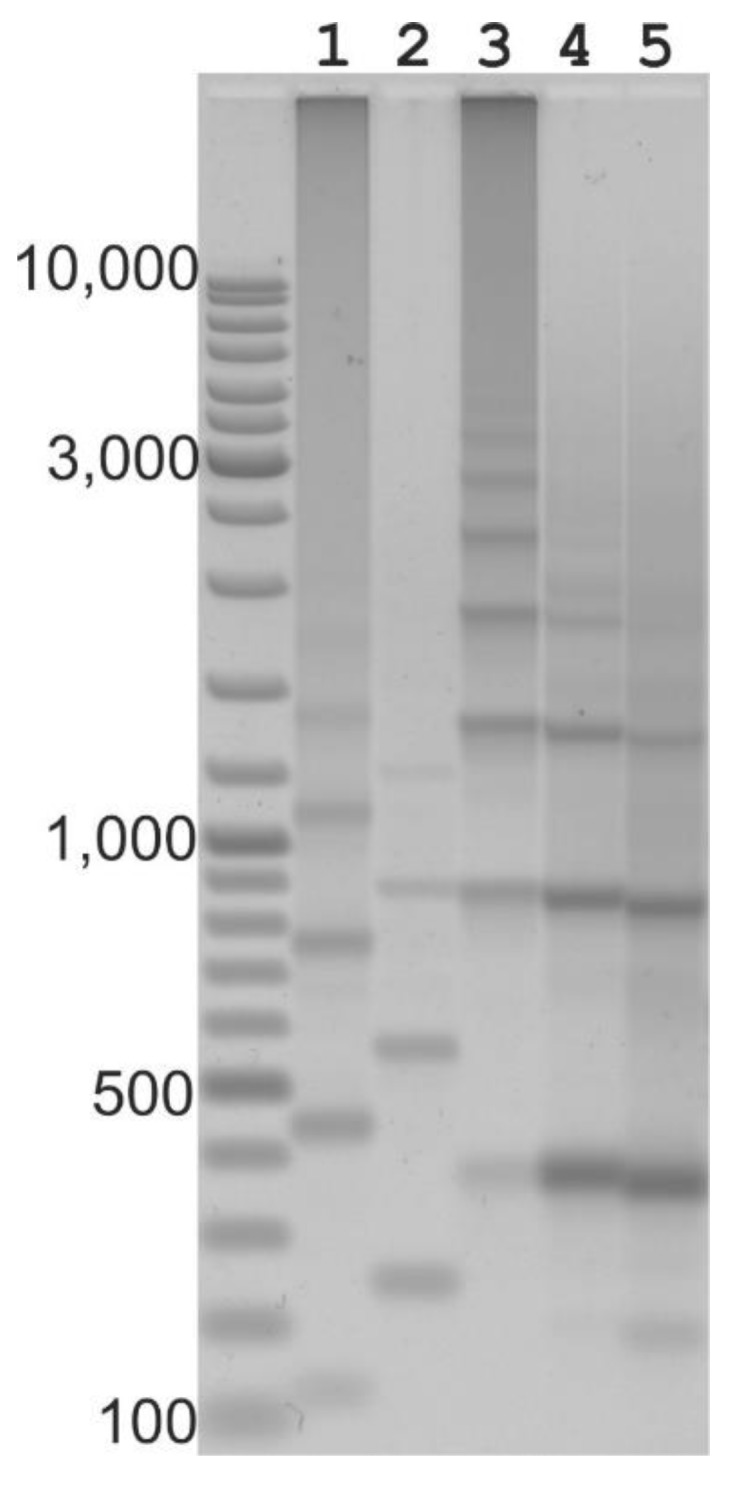
Tandem amplification applied to 5S rRNA and the *Cassandra* retroelement of *Avena sativa*. 1 and 2: 5S rRNA inter-tandem amplification; 1 (primers 1803 and 1804), band sizes (bp), 119, 430, 741, 1052, 1363, 1674, 1985; 2 (primers 2721 and 2722), band sizes 238, 549, 860, 1171, 1482. 3 to 5: *Cassandra* inter-tandem amplifications; 3 (primers 4170 and 4174), band sizes 361, 842, 1323, 1804, 1885, 2366, 2847, 3327, 3809); 4 (primers 3801 and 3802), band sizes 368, 849, 1330, 1811; 5 (primers 3801 and 1032), band sizes 357, 838, 1319, 1800. The predicted lengths for tandems for *Cassandra* retrotransposons, and for the 5S rRNA cluster, are shown in Table 1.

*Cassandra* retrotransposons carry 5S DNA sequences having well conserved RNA polymerase III promoters as part of their LTRs and were found in all vascular plants investigated. Conserved sequences among all *Cassandra* elements were used to identify new copies of this retrotransposon in plant genomes in silico and reveal their presence in plant species where they had not been previously identified. *Cassandra* 5S-rDNA–related sequences contain two conserved regions—box A (RGTTAAGYRHGY) and box C (RRRATRGGTRACY)—separated by 18 nucleotides. Furthermore, a conserved PBS sequence (TGGTATCAGAGC) is located internal to the 5′ LTR in the position standard for LTR retrotransposons, showing as well the typical tRNA complementarity (Figure 1). The *Cassandra* PBS is found 8 bp from 5S rDNA sequence for ferns and 173 bp or longer for *Brassica* species.

*Cassandra* elements were searched for in diverse plant species. The assembled genomes of *Arabidopsis thaliana* (L.) Heynh., *Brachypodium distachyon* (L.) P.Beauv., *Glycine max* (L.) Merr., *Zea mays* L., *Oryza sativa* L., *Sorghum bicolor* (L.) Moench, *Vitis vinifera* L., *Medicago truncatula* Gaertn., *Saccharum hybrid*, *Panicum virgatum* L., and *Solanum lycopersicum* L., as well as the shotgun sequence of *Citrus clementina*, were analyzed. In the *Zea mays* (B73) genome, we discovered the greatest quantity, in total 701 complete *Cassandra* retrotransposons, whereas as the fewest complete elements were found in the genomes of *Arabidopsis thaliana* (4) and *Brachypodium distachyon* (5) (Table 1). These data correspond well with NCBI BLAST data on the copy number of the *Cassandra* retrotransposons for the studied genomes. Analyses of the *Amaranthus palmeri* S.Wats., *Sorghum bicolor*, *Vitis vinifera*, *Medicago truncatula*, *Saccharum hybrid*, *Panicum virgatum* L., *Solanum lycopersicum*, *Garcinia mangostana* L., *Silene latifolia* Poir., *Deschampsia antarctica* É.Desv, *Colobanthus quitensis* (Kunth) Bartl., *Colpodium drakensbergense* Hedberg and I.Hedberg, *Zingeria biebersteiniana* (Claus) P.A.Smirn., *Oryza glaberrima* Steud., and *Oryza minuta* J. Presl genomes revealed novel sequences of *Cassandra* retrotransposons (Table S1). Newly discovered consensus sequences of complete *Cassandra* retrotransposons were submitted to the NCBI database (accession numbers EU140956, EU177767, EU867815, EU882730, FJ975775-FJ975780, HM481419, HM481420, KC686837-KC686839, KM262797, and MT230479).

In silico analyses indicated short tandem arrays of *Cassandra* within many of the investigated plant species. These arrays tended to consist of about three *Cassandra* LTRs and separated by an intervening internal domain sequence. The presence of long tandem arrays in currently available sequence assemblies is limited by the assemblies being based on short-read DNA sequencing approaches, which can tend to pile up tandem repeats. As assemblies based on the newer long-read sequencing techniques such as those of Oxford Nanopore or Pacific Biosciences [32,33] appear, the problem of under-representation of repeat numbers in tandem arrays should decrease.

### 2.2. Long Distance PCR for Complete Tandem Cassandra Cluster Isolation

Due to potential issues in the representation of concatenated repeats in genome assemblies, we investigated the genomic arrangement of *Cassandra* elements by other means. We designed several inverted primer combinations within the internal part of the retroelement close to the PBS or PPT (PolyPurine Tract) sites and within the LTR section of the RNA polymerase III promoters (Table 2, Table S2). Using long-distance PCR, we discovered that *Cassandra* retrotransposons are organized into long arrays of almost identical, tandemly arranged units (Figure 1). *Cassandra* retrotransposons make very good subjects for this type of study. The small size of these mobile elements makes it achievable to see multiple-band amplicon ladders for tandem repeats using even standard PCR.

For tandem repeat amplification and analysis, a pair of inverted primers complementary to the internal part of *Cassandra* and directed away from each other will amplify the flanks of the *Cassandra* elements. If the elements are clustered in arrays, the amplified fragments will contain a series of concatenated *Cassandra* elements. Because smaller fragments are amplified more efficiently during PCR and *Cassandra* is a short TRIM retrotransposon, recovery of such arrays is eased. The genomes of cereals (barley, oats, wheat) contained extended clusters of long tandem arrays of *Cassandra* retrotransposons, and therefore we observed a ladder of amplicons when using long-distance PCR already starting from the 10th PCR cycle. Based on this, the cloning and analysis of tandem repeats were carried out only for amplicons obtained during long-distance PCR at the exponential stage (between 12 and 20 cycles). This allowed us to avoid artefacts due to the concatemerization of the amplicons themselves at the PCR saturation stage. Depending on the pair of PCR primers used, we obtained a ladder of PCR products corresponding to the predicted structure of tandem repeats (Table 2, Table S2). *Cassandra* tandem repeats were found within many different monocots, as well as in the evolutionarily distant dicot species.

**Figure 2:**
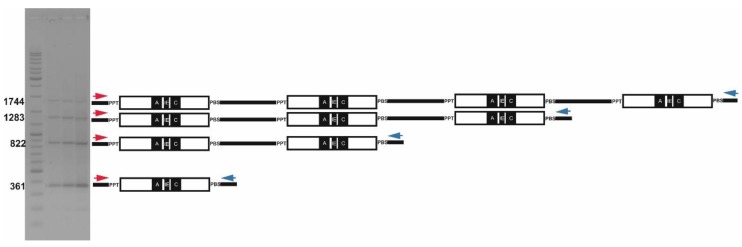
Tandem structure and long-distance PCR (3801-3802), applied to *Cassandra* (*Avena sativa*), multimeric tandem repeats are generated (gel image on the left). Forward primers (red arrows) and reverse primers (blue arrows) respectively.

Tandem-repeat *Cassandra* retrotransposons are organized much the same way that ribosomal genes are structured within eukaryotic genomes. The cellular 5S rRNA genes form tandem units, which are comprised of a 5S rRNA gene and an untranscribed spacer, repeated many times: 5SrRNA-_spacer_-5SrRNA-_spacer_-5SrRNA-_spacer_-5SrRNA. The tandem repeats of *Cassandra* retrotransposons likewise form a sequential arrangement of repeat units, positioned one after the other and comprising LTRs and an internal domain (Figure 2): LTR-_internal_domain_-LTR-_internal_domain_-LTR_-internal_domain_-LTR-_internal_domain_-LTR. For the tandem clusters of 5S rRNA genes, the tandem repeat unit comprises the 5S rRNA gene itself (121 bp) interspersed with an untranscribed spacer. In the case of *Cassandra* retrotransposon arrays, the tandem repeat comprises single LTRs interspersed with internal domains. Given that each *Cassandra* LTR contains a 5S domain with its RNA polymerase III promoter, the *Cassandra* arrays produce a structure highly reminiscent of those composed of 5s rRNA genes: 5S domains interspersed by spacers.

Since amplification for tandem arrays using long-distance PCR is quite effective, we set out to determine the longest product. For this, we used a two-step PCR and increased the temperature of annealing and polymerase elongation to 72 degrees. These conditions contribute to the amplification of long amplicons and inhibit that of short ones (isolated single elements). The number of PCR cycles should not exceed 20, at which point fast saturation and the formation of smeared amplicons begins. Taking into account all these conditions, it was possible for us to use inverted PCR to detect over 12 tandem repeats, which corresponded to tandem arrays comprising clusters of 13 LTRs and 12 internal domains (a ~6 kb PCR fragment). To distinguish a PCR artifact from amplification of genomic tandem arrays, we collected the PCR amplicons from cycles 14 to 23. Artifacts were observed generally when the PCR reached a plateau and concentration of PCR products was sufficient to allow staggered hybridization of the products and their subsequent extension. However, we could easily detect tandem arrays as early as at 14 cycles, when amplification was in the exponential phase.

Favoring the amplification of native tandem arrays rather than artifactual concatemers was the use of inverted primers located within the internal domain *Casandra*, which permits amplification of tandemly duplicated but not single-copy elements. We tested different inverted primer-pair combinations, for which we simulated the expected sizes of ladder of amplicons that would be generated. In each case, we detected the expected amplicon sizes. We did a similar analysis for the 5S rDNA tandem cluster, with a simulation of the expected sizes of the bands within a ladder of amplicons for various inverted primer-pair combinations. We then amplified long tandem arrays for 5S rRNA gene clusters using long-distance inverted PCR with conserved PCR primers (2721 and 622, Table S2) for the 5S rRNA gene for various plant species. The products generated from the 5S rDNA gene cluster using long-distance PCR were as predicted by the simulation. Additionally, we identified long tandem arrays for 5S rRNA genes for newly sequenced genomes. For the scaffold sequence (Brapa_scaffold_36) from the *Brassica rapa* genome (LR031586), an extended region with a length of about 63 kbp was identified that contains a tandem array of 5S rRNA genes. The size of the intergenic spacer between the 5S rRNA gene ranged from 380 to 610 bp and most of the 5S rRNA genes were intact (Table 2).

### 2.3. Cassandra Tandem Repeats in mRNA and Genomic DNA

We looked for the presence of extended tandem arrays of *Cassandra* elements in mRNA transcripts from various sources (leaves, roots, and embryos) for barley, wheat, and other grasses by RT-PCR. We identified tandem *Cassandra* elements in transcripts for the investigated grasses (Table S4). Since mRNAs do not usually exceed 3000 nt, we did not expect to obtain long tandem arrays of *Cassandra* cDNA products. Our data show that the transcribed tandem arrays of *Cassandra* contain from two to five repeat units. In addition, we detected tandem arrays of *Cassandra* for various plant species in the NCBI EST database (Table S3). Thus, from genomic arrays of *Cassandra* elements, multi-element transcripts are produced, presumably by reading through the intervening LTRs and not terminating in the first 3′ LTR as expected in canonical retrotransposon transcription.

### 2.4. Cassandra Secondary Structure

**Figure 3:**
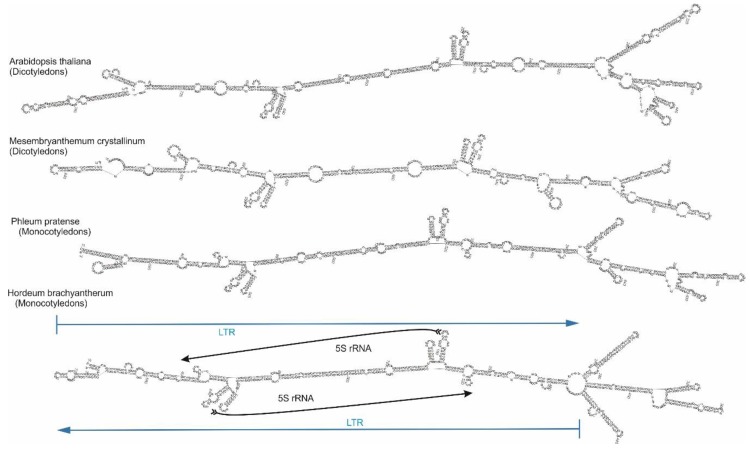
Super-hairpin structure predicted from the sequence of *Cassandra* elements. The whole *Cassandra* element forms a structure at folding, where both LTRs are complementary to each other with the complement region containing a 5S-rDNA sequence. The internal domain of the *Cassandra* element is also self-complementary, and the PBS and PPT domains are located near each other.

We modeled the folding of whole *Cassandra* retrotransposons from different species and compared these. The sequences folded into a conserved super-hairpin structure (Figure 3). The folds are predicted to be functional, based on structural conservation and thermodynamic stability, unlike the reversed sequences for each element. The predicted folds of the *Cassandra* sequences somewhat varied, but they all resembled the canonical super-hairpin structure, which is conserved for all across all plant species, from ferns to grasses, examined thus far. The main structure for all the folds of *Cassandra* elements entails the self-complementarity of both the LTRs as a whole and of the 5S rRNA regions within (Figure 3). Given the secondary structure of individual *Cassandra* elements, transcripts of tandem arrays of *Cassandra* elements are expected to fold into a series of super-hairpin structures comprising the LTR-_internal_domain_-LTR units. The 5S rRNA gene clusters also form self-complementary structures in the same manner as seen for the 5S sequences from the *Cassandra* element.

**Figure 4:**
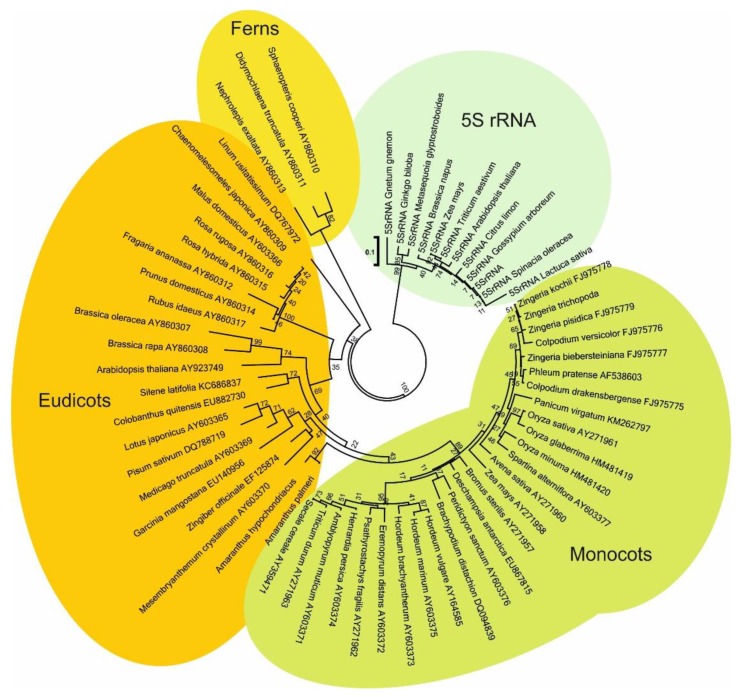
Phylogenetic relationships among *Cassandra* elements by neighbor joining. A maximum likelihood tree with all sequenced *Cassandras* is topologically similar.

For the phylogenetic analysis, we used multiple alignments for the LTRs and internal domains of *Cassandra* elements from each species. Multiple alignment programs cannot identify the structural boundary between the LTR and the internal domain of retrotransposons. Therefore, we performed an alignment between all LTRs, and then separately for the internal domains. These separate alignments were then combined. Phylogenetic analysis of the *Cassandra* elements shows that the patterns of conservation match, as expected, the plant family from which the retrotransposon was isolated (Figure 4). The LTRs, including the PBS sequence, also showed conservation consistent with their parent plant families.

### 2.5. Cassandra Copy Number Variation

Previously, we examined genome size variation and its relationship to *BARE1* LTR retrotransposon accumulation patterns in wild barley (*Hordeum spontaneum*) in the Evolution Canyon (EC), Lower Nahal Oren, Mount Carmel, Israel [34], along a transect presenting sharply differing microclimates [35]. The EC consists of two abutting slopes separated, on average, by 200 m. The “African” south-facing slope (SFS: populations NH, NM, NL, coded as north high, middle, low) receives 200–800% higher solar radiation than that seen by the forested, “European” north-facing slope (NFS: populations SH, SM, SL) [34]. The EC model allows examination of evolutionary mechanisms at a microscale, including biodiversity divergence, adaptation, and incipient sympatric speciation.

We have now examined the copy number variation both of tandem arrays and individual *Cassandra* elements in *H. spontaneum* in the EC. The changes in copy number of tandem arrays and individual elements were measured by two different methods: qPCR and dot blot hybridization. Dot bot hybridization in most cases gave significantly higher copy numbers than did qPCR, although the same trends in EC were seen (Table S3). The higher copy number by dot blot may be due to the higher stringency imposed by mismatches on amplification than on hybridization. The total copy number of *Cassandra* in a handful of barley cultivars were examined by dot blot (Table S4) and tended to be on the high end of numbers seen for the EC *H. spontaneum* accessions. For the following analyses, we consider only qPCR data, as it allows singletons and arrays of *Cassandra* elements to be treated separately. The total copy number within each site varied from 9–22%

*Cassandra* tandem repeats showed great differences in copy numbers between individuals in the accessions, from 200 copies for 59-NH to 1150 copies for 43-NM. The mean numbers of *Cassandra* elements in the EC populations range from 2172 (SH) to 3417 (NM), with the fewest in an individual accession (3-SH) being 1278 on the NFS and the most 5736 (53-NH) on the SFS. Taking the slopes together, the mean total copy number of *Cassandra* was lower at the top of EC (NH and SH together), 2651 elements versus 2947 and 3070 for the lower and middle populations respectively, though given the intrasite variation, the numbers were not significant at *p* < 0.05 by one-way ANOVA. The same trend could be seen for the elements in tandem arrays as for all *Cassandra* together, with the fewest tandem copies at the top of the EC.

Looking at inter-slope differences, populations from the hotter SFS together had significantly more *Cassandra* copies at *p* = 0.003 (Student’s T) than those from the NFS (3182 vs. 2611) (Table S4). The tandemly repeated element count is, though, conflated in the total *Cassandra* copy number as the primers used for singleton *Cassandra* elements amplify also from tandems. The configuration of the PCR primers used to amplify the tandem repeats (Figure 2), however, do not amplify individual elements. When one considers only the single *Cassandra* elements (by subtracting the tandems from the total), the higher number of elements on the SFS becomes still more significant, *p* = 0.0002, (average 2729 SFS vs. 2260 NFS). The reason for the increase in significance is that the number of tandem *Cassandra* elements, follows the opposite trend: the SFS has fewer on average than did the NFS (431 vs. 478), but not significantly so (*p* = 0.15). Commensurate with these observations, the ratio of singleton to tandem *Cassandra* elements was 4.2–4.6 on average for the NFS populations and 5.8–8.1 for the NFS.

### 2.6. Chromosomal Distribution of Cassandra

Using FISH, we investigated the abundance and chromosomal distribution of *Cassandra* retrotransposons in *Aegilops speltoides* Tausch. (2*n* = 2*x* = 14). This is a diploid cross-pollinated species, a wild relative of various wheat species [36] and proposed progenitor of the B-genome of hexaploid wheat [37]. This species serves as an informative model for studying the chromosomal patterns of various types of transposable elements in the plant genome [37,38] and was selected based on the following two criteria: (i) it is a diploid Triticeae grass as is *H. spontaneum* investigated here, and grows in the same region; (ii) the karyotype and repeatome composition of *Ae. speltoides* are well studied [39,40], facilitating comparative analysis.

**Figure 5:**
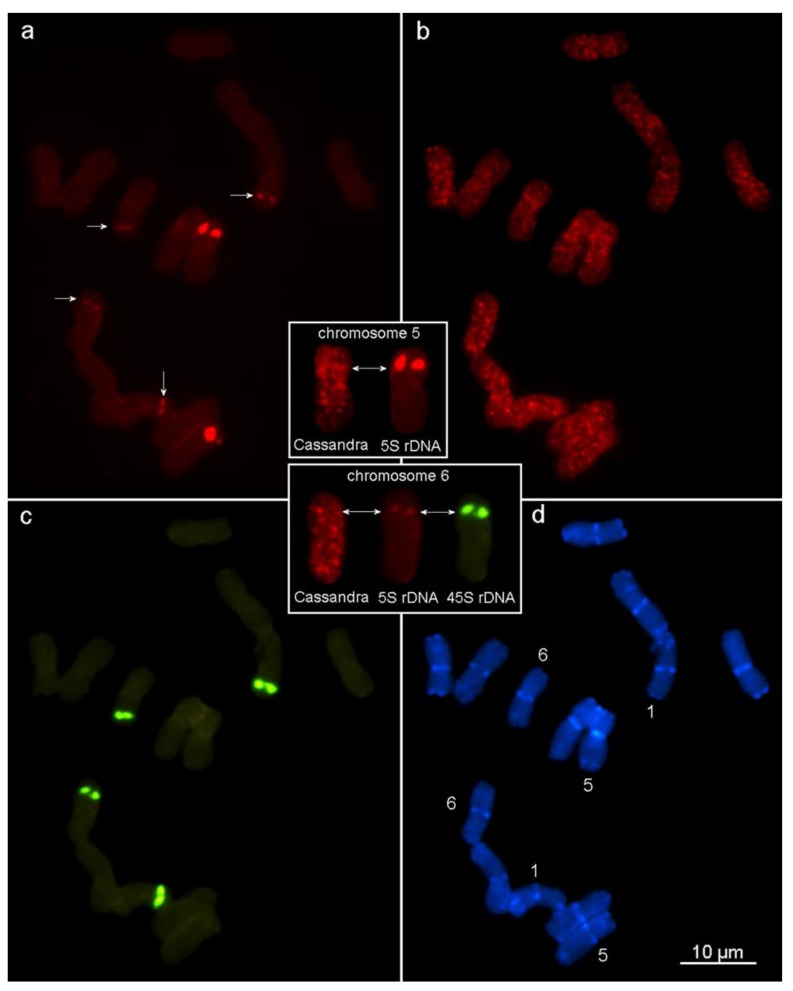
Chromosomal distribution of Cassandra in the Aegilops speltoides Tauch. (2n = 2x = 14) genome. Fluorescent in situ hybridization (FISH) of (**a**) 5S rDNA labeled in red (additional 5S rDNA clusters on chromosomes 1 and 6 are shown with arrows), (**b**) Cassandra retrotransposon (in red), (**c**) 45S rDNA (in green) on metaphase chromosomes of Ae. speltoides, and (**d**) differential staining with DAPI (in blue). Clusters of Cassandra elements have been distinctly observed in euchromatin (**b**), while in distal/terminal heterochromatic regions (**d**) a strong reduction in signal occurs. Notably, distinct clusters of Cassandra elements coincide with regular 5S rDNA blocks on chromosome 5 as well as with additional blocks on chromosomes 1 and 6 that normally carry 45S rDNA gene clusters (arrows on the enlarged chromosomes 5 and 6).

The cytogenetic analysis displayed certain features in the chromosomal patterning of the *Cassandra* retrotransposon in *Ae. speltoides*. The FISH experiments revealed widely spread mini clusters in euchromatin and prominent TE clustering in pericentromeric and subtelomeric chromosomal positions (Figure 5). The pattern of *Cassandra* distribution was chromosome specific and was similar in homologous pairs of chromosomes. Chromosome 5 carried large *Cassandra* clusters adjacent to 5S rDNA blocks, and chromosomes 1 and 6 carry TE clusters coinciding with additional 5S rDNA clusters in the 45S rDNA (nuclear organizer regions) clusters (Figure 5, small boxes).

## 3. Discussion

We previously identified large, structurally uniform retrotransposon groups, in which no member contains the *gag*, *pol*, or *env* internal domains. These non-autonomous groups have been named LARDs and TRIMs [25,30], and they specify well-conserved RNA secondary structures. The LARD and TRIM phylogenies mirror those of their host organisms [25]. Because of the lack of protein coding capacity, these groups are replicationally non-autonomous, even if they are transcriptionally active. *Cassandra* elements belong to the TRIM group. *Cassandra* elements universally carry conserved 5S RNA sequences and associated RNA polymerase (pol) III promoters and terminators in their long terminal repeats (LTRs).

Here, we explored the sequence, structure, genomic organization, and genome dynamics of *Cassandra* elements in a wide range of plants, with a focus on Triticeae grasses. *Cassandra* was found in the genomes of all species investigated. We demonstrated that *Cassandra* is transcribed, consistent with its presence in EST databases. Fold modeling of *Cassandra* transcripts indicates a conservation of secondary structure across the species investigated. The key elements in the secondary structures are stems formed by the LTRs and their component 5S domains. On the DNA level, the degree of sequence conservation in *Cassandra* generally follows the phylogenetic relationships and evolutionary distance of their host species.

We previously observed a similar genetic and phylogenetic relationship of sequences of mobile elements with their host genome. For example, the *BARE1* RLC retrotransposon was detected both in closely related species and in genetically distant ones [41,42]. Likewise, *Sukkula*, a non-autonomous LARD retrotransposon, has been identified among a large number of grasses species [25]. Therefore, using the sequences of mobile elements, it is possible to identify the evolutionary relationship of one plant species to other species; this has been a relatively underexploited approach [25,30,43,44,45]. Nevertheless, horizontally transferred (HT) transposable elements may be fairly frequent in various genomes [46,47]. Depending on when the HT event occurred, such elements would distort the estimated divergence time in a phylogeny by a lesser or greater extent. This complication could be controlled for by carrying applying jackknife analysis to determine statistical support for the phylogeny, as has been done for microbial genomes [48].

Strikingly we found that many *Cassandra* retrotransposons are organized in long arrays of almost identical, tandemly arranged units comprising alternating LTRs and internal domains, much the same way that ribosomal genes are structured within eukaryotic genomes, within all studied plant species, consistent with those earlier observed [49,50]. We observed blocks of *Cassandra* tandem repeats on all chromosomes investigated, with up to 12 repeats per array. However, experimental detection of intact long arrays is hindered by the amplification efficiency of PCR; the occurrence of such repeats in genome assemblies moreover is limited, as it is for rDNA repeats, by sequencing and assembly methods. The 5S rDNA domains of the *Cassandra* LTRs set up a pattern very reminiscent in spacing of the 5S-spacer-5S pattern of ribosomal rDNA repeats. By some hybridization approaches, the *Cassandra* 5S arrays could be indistinguishable from the ribosomal repeats.

The widespread occurrence of tandem arrays of *Cassandra* raises two questions: one of how they are formed; the other of what, once they are formed, controls their repeat dynamics. The process of replication and integration of retrotransposons normally leads to single copies being integrated [2]. By one mechanism, a subsequent intrachromosomal recombination between the two most proximal LTRs would generate an initial array of three LTRs and two internal domains [51]. While the process can be deleterious because any intervening gene would thereby be lost, as we have documented [52], there appear to be at least 4600 such structures for the retrotransposon *BARE1* in the barley genome [51]. An alternative mechanism, however, involves template switching during the reverse transcription of gRNA, a process we have also documented [53]. The compactness of *Cassandra* would make the capacity of the virus-like particle less of a limiting factor for a concatenated cDNA. The concatenated *Cassandra* transcripts detected here, moreover, might favor further extension during reverse transcription. The two proposed mechanisms are not mutually exclusive.

Once a short tandem array of *Cassandra* elements is present in the genome, the numbers of repeats may dynamically fluctuate through a process similar to that for 5S rRNA genes, involving inter-chromosomal unequal crossing-over or chromatid exchange [15]. Although sister-chromatid and inter-chromosomal crossing-over can have similar effects on copy-number distribution, they would have very different consequences for the homogenization of the elements involved. If copy numbers fluctuate via ectopic (i.e., non-allelic homologous) exchanges, the copies of each repeating unit should be different. However, we observe no such differences. Some *Cassandra* tandems have incomplete terminal repeat units that are smaller in size than the other units. A key feature of this mechanism is these types of tandems appearing to have incomplete or truncated repeat units at the end of the repeat array. Mechanisms of slipped-strand mispairing, as well as of *Cassandra* unit conversion or unequal crossing over during meiosis replication may cause gain or loss of a copy of the region flanked by such small direct repeats. But these mechanisms may explain of only part of all *Cassandra*-derived tandems. The remaining tandems are not flanked by direct repeats or incomplete terminal repeats.

The eco-genomic patterns presented here for the two sharply divergent slopes of the EC, where great variation in the number of both singleton and tandem *Cassandra* elements in the predominantly self-pollinated *H. spontaneum* were found, may shed light on the mechanism of *Cassandra* gain and loss. The hotter, drier SFS had both more *Cassandra* copies overall and more as singletons, but fewer in tandem arrays, at all three stations, compared to the NFS. The ratio of copies in singletons versus tandem arrays was greatest at the driest and hottest station, NH-7.1. A very similar phenomenon was found for the *BARE1* at the same EC stations: more full-length elements and fewer solo-LTRs [35]. *BARE1* is an autonomous RLC (*Copia*) LTR-retrotransposon with long (1.8 kb) LTRs and a 6 kb protein-coding internal domain, while *Cassandra* has a very small stuffer fragment for an internal domain and exceptionally short LTRs. Nevertheless, for both systems, abiotic stress appears to both drive higher copy numbers and suppress LTR: LTR intra-chromosomal recombination. For *BARE*, the recombination generates solo LTRs, but for *Cassandra*, long arrays of tandem repeats.

In peripheral or marginal populations of *Aegilops speltoides*, a diploid predominantly cross-pollinated but self-compatible species, an increase in the recombination frequency is seen when the stressed population shifts its mating system [15,54]. Significant temporal fluctuation in the copy numbers of *Cassandra* retroelements was detected in ontogenesis in individual genotypes of *Ae. speltoides* form marginal stressed populations of Kishon [15]. The population is characterized by high heteromorphy and possesses a wide spectrum of chromosomal aberrations, supernumerary B chromosomes, and appearance of additional 5S ribosomal DNA (rDNA) sites at novel chromosomal locations [55]. It was discovered that the *Cassandra* copy number oscillated between 1.5- to 2.5-fold between gametophytes and sporophytes in original plants from this population and the selfed progenies [15]. Moreover, in *Ae. speltoides*, large clusters of *Cassandra* retroelements coincide with regular 5S rDNA blocks on chromosome 5 and with additional 5S rDNA clusters arising in the 45S rDNA loci on chromosomes 1 and 6 (Figure 5). Dispersal clusters of *Cassandra* elements in euchromatin, along with other types of tandem repeats could be considered as an additional target for homologous and ectopic recombination in somatic and meiotic cells in [8,10]. We did not, however, assess the relative balance of singleton and tandem *Cassandra* elements in geographic gradients of *Ae. speltoides*.

In summary, *Cassandra* represents a remarkably dynamic genomic system entailing replication by means of (unknown) autonomous retrotransposon partners, generation and fluctuation in tandem array copies, and a 5S rDNA domain of unknown function. The general features and dynamism of *Cassandra* appear to be conserved across the plant kingdom. Given the stress-driven variability established here in the Triticeae grasses, *Cassandra* elements likely play an important role in genome dynamics in many other clades within the plant kingdom.

## 4. Materials and Methods

### 4.1. Plant Material

Analyses were conducted on various members of the Poaceae (grasses; monocots): *Bromus sterilis L*; *Agropyron cristatum* (L.) Gaertn.; *Amblyopyrum muticum* (Boiss.) Eig.; *Australopyrum retrofractum* (Vickery) Á.Löve; *Australopyrum velutinum* (Tzvelev) Á.Löve; *Comopyrum comosum* (Sibth. and Smith) Á.Löve; *Crithodium monococcum* (L.) Á.Löve; *Crithopsis delileana* (Schult. and Schult.f.) Roshev; *Dasypyrum vilosum* (L.) Borbás, Term.; *Eremopyrum distans* (K.Koch) Nevski; *Eremopyrum triticeum* (Gaertn.) Nevski; *Festucopsis serpentinii* (C.E.Hubb.) Melderis; *Henrardia persica* (Boiss.) C.E.Hubb.; *Heteranthelium piliferum* (Sol.) Hochst; *Hordeum brachyantherum* ssp. Californicum (Covas and Stebbins) Bothmer, N. Jacobsen and Seberg.; *Hordeum erectifolium* Bothmer, N.Jacobsen and R.B.Jørg.; *Hordeum marinum* ssp. Gussoneanum (Parl.) Thell.; *Hordeum murinum* ssp. Glaucum (Steud.) Tzvelev; *Peridictyon sanctum* (Janka) Seberg, Fred. and Baden; *Psathyrostachys fragilis* ssp. Fragilis (Boiss). Nevski; five diploid *Aegilops* species (SS-genomes, 2*n* = 2*x* = 14) belonging to sect. *Sitopsis*, *Aegilops speltoides* Tausch, *Ae.sharonensis* Eig, *Ae. longissima* Schweinf. and Muschl., *Ae. searsii* Feldman and Kislev ex Hammer, and *Ae. bicornis* (Forsk.) Jaub. and Sp.; diploid wheats *Triticum urartu* Thum. Ex Gandil.; *T. boeoticum* Boiss. (A^u^A^u^- and A^b^A^b^-genomes, respectively, 2*n* = 2*x* =14, wild progenitors of the A-genome of allopolyploid wheats); allopolyploid wheats *T. dicoccoides* (AABB-genome, 2*n* = 4*x* = 28) and *T. timopheevii* (A^t^A^t^GG-genome, 2*n* = 4*x* = 28). From the eudicots, we examined *Lotus corniculatus* L., *Medicago truncatula* Gaertner (var. longeaculeata Urban), *Mesembryanthemum crystallinum* L., *Prunus domestica* L., *Malus domestica* Borkh., *Chaenomeles japonica* Lindl. ex Spach, *Rubus idaeus* L., *Rosa rugosa* Thunb., and *Fragaria x ananassa* Royer. The ferns *Nephrolepis exaltata* (L.) Schott, *Sphaeropteris cooperi* (Hook. ex F.Muell.) R.M.Tryon (*Cyathea cooperi* (F.Muell.) Domin), were gifts of the University of Helsinki Botanic Garden (Table S1).

### 4.2. Computation Analysis and Alignment

Sequence analyses using EMBOSS and the Multiple Sequence Alignment were run on the EMBL-EBI (https://www.ebi.ac.uk/Tools/msa/) online platform. The BLAST searches for *Cassandra* sequence similarity were made online at the National Center for Biotechnology Information web site (https://blast.ncbi.nlm.nih.gov/Blast.cgi). The cellular 5S rRNA sequences were retrieved for analysis from a dedicated database (http://combio.pl/rrna/). We aligned the *Cassandra* 5S rDNA domains first within plant families and then realigned each set with the aligned ribosomal 5S rDNA set. Finally, a global alignment was carried out. RNA folding prediction was carried out with the MFOLD web server (http://unafold.rna.albany.edu/?q=mfold/) [56] at a folding temperature of 17 °C. This was chosen to reflect ambient conditions for plants.

### 4.3. Phylogenetic Analyses and Tree Building

Phylogenetic analysis was performed using UGENE software (http://ugene.net/) [57] and (https://ngphylogeny.fr/) [58]. Trees were constructed by the Neighbor-Joining method and compared with those from the Maximum Parsimony and Maximum Likelihood methods. All positions containing alignment gaps and missing data were eliminated only in pairwise sequence comparisons (pairwise deletion option). 

### 4.4. In Silico Query for Cassandra in Plant Genomes

In silico searches for the LTR retrotransposon *Cassandra* and tandem arrays in plant genomes was performed by FastPCR software using the “Linked (Associated) search” in the in silico PCR tool. Search criteria were based on the distance between sequences and their match similarity, which starts with fast fuzzy string searching [59,60]. A method called linked (associated) searching allows advanced searching of sequence-template binding sites in a variety of scenarios, including that of in silico PCR [61]. The “Linked (Associated) search” tool for conserved *Cassandra* sequences was used for detection of *Cassandra* retrotransposons in plant genome sequences where they had not been previously identified. *Cassandra* universally carries conserved 5S rDNA sequences in each LTR. The 5S domains in turn each have an RNA polymerase III promoter and two conserved regions, boxA (RGTTAAGYRHGY) and boxC (RRRATRGGTRACY), separated by 18 nucleotides. Furthermore, *Cassandra*, like other LTR retrotransposons, contains a conserved primer binding site (PBS; TGGTATCAGAGC) internal to the 5′ LTR. In ferns, the PBS is located 8 nt from the 5S rDNA sequence whereas in *Brassica* species it is up to 173 bp away. Therefore, we used following linked-sequences query in FASTA format to search both fern and seed plant genomes for Cassandra sequences: >Cassandra RGTTAAGYRHGY[15–25]RRRATRGGTRACY[5–200]TGGTATCAGAGC.

The following genomes were searched with the above query: *Arabidopsis thaliana*, *Brachypodium distachyon*, *Glycine max*, *Zea mays*, *Oryza sativa*, *Sorghum bicolor*, *Vitis vinifera*, *Medicago truncatula*, *Saccharum hybrid*, *Panicum virgatum*, *Solanum lycopersicum*, *Citrus clementina* and other newly sequenced plant genomes (Table S1).

In addition, we performed the “Linked (Associated) search” tool to identify long tandem arrays for *Cassandra* within all studied plant species. The search for long tandem arrays of *Cassandra* were specified as: (RGTTAAGYRHGY[15–25]RRRATRGGTRACY(5–200)TGGTATCAGAGC)n, where n is the amount of tandem arrays, n = 1 for a single copy of Cassandra and >1 for a tandem array: >Cassandra_tandem RGTTAAGYRHGY[15–25]RRRATRGGTRACY(5–200)TGGTATCAGAGC[200–1000]RGTTAAGYRHGY[15–25]RRRATRGGTRACY[5–200]TGGTATCAGAGC.

### 4.5. Primer Design

The sequences of *Cassandra* retrotransposon accessions were aligned and conservation assessed with the multiple alignment procedure of Multain [62]. For *Cassandra*, LTRs and an internal part of the retrotransposon showed variability, but certain regions were relatively conserved. The conserved segments of the LTR were used for the design of PCR primers. PCR primers were designed, using FastPCR software v.6.7 [59,60], also to match the internal part of the retrotransposon sequence near to either its 5′ or 3′ end, with the primer oriented so that the amplification direction is towards the nearest end of the LTR (Table S2). Several inverted primers were designed at both ends of the *Cassandra* LTR in order to compare the efficiency and reproducibility of amplification. The sequences of the primers are shown in Table S2. The chosen primers matched the motifs sufficiently conserved in the retrotransposons to allow amplification of the great majority of targets in the genome. For 5S rRNA genes, multiple alignments were made on the sequence accessions to identify the conserved segments used for the design of PCR primers (Table S2).

### 4.6. DNA Extraction

DNA was isolated from leaves using the CTAB extraction protocol described at http://primerdigital.com/dna.html with RNAse A treatment. The detailed protocol for DNA isolation was submitted to https://www.protocols.io/ (DOI:10.17504/protocols.io.z2jf8cn) [63]. DNA samples were diluted in 1 × TE buffer and the DNA quality was checked electrophoretically as well as spectrophotometrically with a Nanodrop apparatus (Thermo Fisher Scientific Inc., Waltham, MA, USA).

### 4.7. Long-Distance Inverted PCR to Isolate Cassandra Tandems

*Cassandra* tandem arrays were identified and extracted using long-distance PCR with inverted primers from the internal domain of the retrotransposon. The amplifications were carried out with low numbers of PCR cycles (12–20) to avoid formation of non-specific PCR products, assuming a high copy number for the retroelement of interest, as earlier described [64]. Several primer pairs were designed for each identified element, oriented away from each other as for inverse PCR (Table S2). Inverted primers of 20–24 nt were designed from the internal domain with high Tm (>55 °C). This allows annealing and polymerase extension in one step at 70–75 °C, thereby increasing the efficiency of the amplification of long fragments. Long distance PCR was performed with LongAmp™ Taq DNA Polymerases (New England Biolabs, Ipswich, Massachusetts, USA). The 25 μL reaction volume contained: 1 × LongAmp™ *Taq* buffer, 25 ng DNA, 300 nM of each primer, 200 μM dNTP, and 1 μL LongAmp™ *Taq* DNA Polymerase. The reaction cycle consisted of a 2-min initial denaturation step at 95 °C; 15–22 cycles of 15 sec at 95 °C, 1 min at 70 °C, and 4 min at 72 °C; a final extension of 5 min at 72 °C.

### 4.8. Gel Electrophoresis

The PCR products were separated by electrophoresis at 70 V for 3 h in a 1.3% agarose gel (Wide Range; SERVA Electrophoresis GmbH, Heidelberg, Germany) with 0.5 × TBE electrophoresis buffer. Gels were stained with EtBr and scanned using an FLA-5100 imaging system (FUJI Photo Film GmbH; now FUJIFILM Europe GmbH, Heidelberg, Germany) with a resolution of 50 µm. Fragment size was determined with the aid of a DNA ladder for electrophoresis, GeneRuler™ DNA Ladder Mix (Thermo Fisher Scientific, Waltham, MA, USA), 100–10,000 bp range.

### 4.9. Cloning PCR Fragments

PCR amplicons were separated on 1.3% agarose gels, gel-extracted using a QIAEX II Gel Extraction Kit (Qiagen, Hilden, Germany) or similar, permitting isolation of DNA fragments from 40 bp to 50 kb from the gel. DNA fragments were cloned using a PCR product TA cloning kit, TOPO^®^ TA Cloning^®^ Kit (pCR^®^2.1 plasmid vector) with transformation into TOP10 *E. coli* (Invitrogen, Carlsbad, CA, USA). Transformed colonies containing insert-bearing plasmids were detected using white-blue screening on selective growth medium containing ampicillin, X-Gal, and IPTG. Positive colonies were tested for the presence of cloned PCR products by PCR with universal pUC primers (forward and reverse M13 primers), followed by separation and visualization of PCR products on agarose gels. Plasmid DNA was extracted using GeneJET Plasmid Miniprep Kit (Thermo Fisher Scientific, Waltham, Massachusetts, USA), and sequencing PCRs performed using an ABI3700 Bioanalyser (Applied Biosystems, Foster City, CA, USA). The *Cassandra* sequences reported in this paper were deposited in GenBank as various accessions (Table S1).

### 4.10. Quantitative Real-Time PCR and Relative Quantification

The copy number of singleton *Cassandra* elements for the *H. vulgare* and *H. spontaneum* genomes was determined by PCR amplification for intact *Cassandra* element amplicons, which included both LTRs and the entire internal domain of the retrotransposon. Primers were designed to match conserved regions in the LTRs of *H. vulgare Cassandra* elements, comprising forward primer 784 (location in LTR, nt 161 to 182) and reverse primer 977 (173 to 153), shown in Table S2. To amplify *Cassandra* tandem arrays specifically, this approach is not applicable. To amplify the minimum unit of *Cassandra* tandem arrays (two concatenated elements), it is necessary to obtain a PCR product between two tandem elements across the intervening LTR from the internal domains of the retrotransposon. Therefore, conserved inverted primers matching the internal domain of *H. vulgare Cassandra* elements were used, comprising reverse primer 981 (308←327) and forward primer 982 (428→448), shown in Table S2, for this approach. Only in this case, the use of inverted PCR from the internal domain of the retrotransposon, can a PCR product corresponding specifically to tandem arrays of *Cassandra* elements be obtained.

Quantitative PCR was performed in a LightCycler^®^ 480 System (Roche, Basel, Switzerland) in 384-well plates. The 10 μL reaction volume contained: 1 × Phire^®^ buffer, 10 ng DNA, 300 nM each primer, 200 μM dNTP, 1U Phire^®^ Hot Start II DNA Polymerase (Thermo Scientific, Waltham, Massachusetts, USA), and 0.5 × SYBR Green I (Cambrex Bio Science Rockland, Inc., Rockland, USA). The amplification program consisted of 98 °C, 2 min; 25 cycles of 10 sec at 98 °C, 10 sec at 60 °C, 10 sec at 72 °C (fluorescence was monitored during this step). All DNA samples were repeated four times per 384-well plate. Relative quantification was used to compare the cycle threshold (Ct) of unknown samples against a standard curve of a sample with known copy numbers. A standard curve was developed by plotting the logarithm of known concentrations (2-fold dilution series from 10 ng per 10 μL reaction volume solution) of the reference sample, *H. vulgare* (cv. Bomi), in which concentration was determined spectrophotometrically after RNase treatment and purification, against the cycle threshold (Ct) value. The Ct value is inversely proportional to the log of the initial concentration, so that the lower the Ct value, the higher the initial copy number of the TE. The Ct values were automatically selected on the ABI PRISM 7000 for each assay type and the data were exported into Microsoft Excel for further analysis.

The efficacy of the PCR was determined by recording a standard curve using sequential dilutions of the *H. vulgare* DNA. A standard curve with a correlation coefficient of about 0.99 and a slope of about −3.3 on a semi-logarithmic plot (a tenfold different concentration of the target gene should result in Ct values with a difference of 3.3) was sought. The efficiency of qPCR and reproducibility of the results did not depend on the length of amplified fragments. The correlation coefficient was close to ideal (0.97 to 0.99) for all primer combinations that were used in qPCR.

### 4.11. Cassandra TRIM Element Copy Number Estimation by Dot Blot

For *Cassandra* copy number determinations by dot blot hybridization, a previously described approach was used [35]. Dot blots were prepared with multiple replicates by using 1 ng or 10 ng of genomic DNA per sample and cross-linked under UV light. For the *Cassandra* probe, a PCR fragment (using primers 784 and 975) was amplified from barley cv. Bomi. This generated a 388-bp probe, which extends from the 5′ LTR beyond the 5S promoter through the internal region to the 3′ LTR and terminates before the 5S promoter of the 3′ LTR. Thus, the part of the *Cassandra* 5S most conserved with the 5S rDNA genes was not part of the probe, avoiding cross-hybridization.

Probes were random-primed (GE Healthcare Amersham™ Megaprime™ DNA Labeling System) and ^32^P-labeled. Filters were hybridized in 50% formamide, 1.25× standard saline phosphate/EDTA (0.18 M NaCl/10 mM phosphate, pH 7.4/1 mM EDTA), 5× Denhardt’s reagent, 0.5% SDS, and 20 μg/mL sonicated herring sperm DNA overnight at 42 °C. Hybridized filters were washed successively with 2× SSC, 0.1% SDS (10 min, 25 °C); twice in 2× SSC, 0.1% SDS (10 min, 65 °C); and once in 0.2× SSC (20 min, 65 °C). Bound radiation was quantified by exposure to a PhosphorImager screen for 45 min followed by scanning on an FLA-5100 imaging system (FUJI Photo Film GmbH). The same filter was hybridized with the *Cassandra* probe described above. Hybridization response to the genomic DNA was corrected to the average value for the *Cassandra* PCR product hybridization response and the relative copy number calculated. The absolute copy number was calculated from the hybridization response of the genomic DNA compared with the control for the *Cassandra* PCR product: copies(ng) = (genomic cpm)/(ng) × (PCR fragment copies)/(PCR fragment cpm). Copies (ng) were converted to copies (genome) by using the *H. vulgare* genome size (where 100 ng barley genomic DNA equals 50 pg of 388 bp *Cassandra* PCR fragment, so at 6,185 copies per 4.8 × 10^9^ bp (0.05% genomic DNA).

### 4.12. Probe Labeling, In Situ Hybridization, and Differential Staining

*Ae. speltoides* plants taken directly from the population Kishon (Kishon River, Haifa Bay area, Israel) were used for investigation of the *Cassandra* chromosomal pattern. For the fluorescent in situ hybridization (FISH) experiments, cytological slides of individual seedling shoot apical meristems containing well-spread chromosomes were used. The chromosome spreads, DNA probe labeling, and FISH procedures were conducted as previously described [55]. The PCR probe corresponding to *Cassandra* (AY271963) was labeled with Cy-3 (Amersham, London, United Kingdom) and used as a probe for FISH. For the second round of FISH on the same chromosomal spread, simultaneous localization of 45S rDNA and 5S rDNA regions—as visualized by the pTa71 [65] and As5SDNAE [66] probes—were used respectively. Probe pTa71 was labeled with fluorescein-12-dUTP (Roche). The As5SDNAE probe was labeled with Cy-3 (Amersham, United Kingdom). The AT-specific 4′,6-diamidino-2-phenylindole (DAPI) fluorochrome was used for differential staining to reveal AT-enriched heterochromatin patterns in the *Ae. speltoides* genome [55]. The slides were examined on a Leica DMR microscope using a DFC300 FX CCD camera.

## Figures and Tables

**Table 1:** Total numbers of hits returned by the “Linked (Associated) search” of several eukaryotic genomes for the highly conserved sequence of the plant LTR retrotransposon *Cassandra*.

Genome	Size in Mb	Predicted Copy Number
*Arabidopsis thaliana*	126	4
*Brachypodium distachyon*	275	5
*Oryza sativa*	380	69
*Medicago truncatula*	391	32
*Vitis vinifera*	420	20
*Sorghum bicolor*	545	64
*Glycine max*	965	30
*Zea mays*	2100	701
*Homo sapiens*	3140	0

**Table 2:** In silico PCR prediction for PCR fragments lengths for *Cassandra* tandem arrays in various plant species.

Plant Species	Formula for Expected Ladder Lengths of PCR Fragments for *Cassandra* Tandems	Forward Primer ID	Reverse Primer ID
*Hordeum vulgare L.*	361 + (461)_n_	981	982
*Hordeum vulgare L.*	374 + (461)_n_	2259	982
*Triticum durum Desf.*	326 + (460)_n_	1032	982
*Triticum durum Desf.*	360 + (460)_n_	981	982
*Triticum durum Desf.*	422 + (460)_n_	1032	2258
*Secale cereal L.*	326 + (460)_n_	1032	982
*Secale cereal L.*	348 + (460)_n_	981	530
*Secale cereal L.*	360 + (460)_n_	981	982
*Secale cereal L.*	422 + (460)_n_	1032	2258
*Avena sativa L.*	357 + (481)_n_	1032	3801
*Avena sativa L.*	361 + (481)_n_	4170	4174
*Avena sativa L.*	368 + (481)_n_	3802	3801
*Avena sativa L.*	494 + (481)_n_	784	977
*Zea maize L.*	431 + (508)_n_	1032	2263
*Zea maize L.*	501 + (508)_n_	2262	2263
*Spartina alterniflora Loisel.*	358 + (537)_n_	1032	3803
*Spartina alterniflora Loisel.*	408 + (537)_n_	1032	3804
*Brachypodium distachyon (L.) P.Beauv.*	314 + (415)_n_	1032	2261
*Brachypodium distachyon (L.) P.Beauv.*	414 + (415)_n_	2260	2261
*Medicago truncatula Gaertn.*	468 + (459)_n_	2070	2071
*Lotus corniculatus L.*	473 + (464)_n_	2070	2071
*Malus domestica Borkh.*	364 + (377)_n_	921	1611
*Vaccinium sp.*	414 + (418)_n_	2016	622
*Garcinia mangostana L.*	525 + (509)_n_	2495	2496
*Silene latifolia Poir.*	473 + (491)_n_	623	629
*Nephrolepis exaltata (L.) Schott*	380 + (378)_n_	1118	1120
5S rRNA *Brassica rapa* (LR031586)	510..527 + (503..520)_n_	2721	622

## Data Deposition

The sequences reported here have been deposited in the GenBank database (accession nos. AY603371, AY923749, AY271960, DQ094839-DQ094843, AY860307, AY860308, AY271957, FJ975775, AY860309, EU882730, FJ975776, EU867815, AY860311, AY603372, AY860312, EU140956, EF125870, AY603374-AY603375, AY164585, DQ767972, AY603364-AY603370, AY860313, HM481419, HM481420, AF538604, AF538611, AY271961, AY603376, AF538603-DQ788719, EF125876, EF125877, AY860314, AY271962, EF125871, AY860315-AY860317, KC686838, KC686839, EF125872, EF125873, AY359471, KC686837, EU177767, AF538605, EF125875, AY603377, AY860310, AY271963, DQ673669, AF538618, AY271958, AY271959, FJ975777-FJ975779, EF125874, MT230479).

## Supplementary Materials

Supplementary materials can be found at https://www.mdpi.com/1422-0067/21/8/2931/s1.

## Abbreviations

EC	Evolution Canyon
LARDs	LArge Retrotransposon Derivatives
LTR	long terminal repeat
MITEs	Miniature Inverted-repeat Transposable Elements
NFS	North-Facing Slope
PBS	Primer Binding Site
PPT	PolyPurine Tract
RLC	LTR retrotransposon of superfamily *Copia*
RLX	LTR retrotransposon
TE	transposable element
TRIMs	Terminal Repeats in Miniature
