# Supplementary material for: Long Tandem Arrays of Cassandra Retroelements and Their Role in Genome Dynamics in Plants
Source: Int J Mol Sci. 2020 Apr 22;21(8):2931. doi: 10.3390/ijms21082931 (PMC7215508; doi:10.3390/ijms21082931)
Supplement: Supplementary file 1 [file ijms-21-02931-s001.pdf]

Supplementary Information

Long Tandem Arrays of *Cassandra* Retroelements and Their Role in Genome Dynamics in Plants

Ruslan Kalendar [1,2,*], Olga Raskina [3], Alexander Belyayev [4] and Alan H. Schulman [5,6,*]

1 Department of Agricultural Sciences, University of Helsinki, P.O. Box 27 (Latokartanonkaari 5), FI-00014 Helsinki, Finland
2 RSE "National Center for Biotechnology", Korgalzhyn Highway 13/5, 010000 Nur-Sultan, Kazakhstan
3 Institute of Evolution, University of Haifa, Mount Carmel, Haifa 31905, Israel; raskina@research.haifa.ac.il
4 Laboratory of Molecular Cytogenetics and Karyology, Institute of Botany of the ASCR, Zámek 1, CZ-252 43 Průhonice, Czech Republic; alexander.belyayev@ibot.cas.cz
5 Natural Resources Institute Finland (Luke), Latokartanonkaari 9, FI-00790 Helsinki, Finland
6 Institute of Biotechnology and Viikki Plant Science Centre, University of Helsinki, P.O. Box 65, FI-00014 Helsinki, Finland
* Correspondence: ruslan.kalendar@helsinki.fi (R.K.); alan.schulman@helsinki.fi (A.H.S.)

Supplementary Table S1. Plant species analysed and corresponding *Cassandra* accessions.

Plant species	*Cassandra* Genbank accession
Amaranthus palmeri	MT230479
Amblyopyrum muticum	AY603371
Arabidopsis thaliana	AY923749
Avena sativa	AY271960
Brachypodium distachyon	DQ094839-DQ094843
Brassica oleracea	AY860307
Brassica rapa	AY860308
Bromus sterilis	AY271957
Colpodium drakensbergense	FJ975775
Chaenomeles japonica	AY860309
Colobanthus quitensis	EU882730
Colpodium versicolor	FJ975776
Deschampsia antarctica	EU867815
Didymochlaena truncatula	AY860311
Eremopyrum distans	AY603372
Fragaria x ananassa	AY860312
Garcinia mangostana	EU140956
Glycine max	EF125870
Henrardia persica	AY603374
Hordeum brachyantherum	AY603373
Hordeum marinum	AY603375
Hordeum vulgare	AY164585
Linum usitatissimum	DQ767972
Lotus japonicus	AY603364, AY603365
Malus x domestica	AY603366-AY603368
Medicago truncatula	AY603369
Mesembryanthemum crystallinum	AY603370
Nephrolepis exaltata	AY860313
Oryza glaberrima	HM481419
Oryza minuta	HM481420
Oryza sativa	AF538604, AF538611, AY271961
Peridictyon sanctum	AY603376
Phleum pratense	AF538603, AF538606-AF538617
Pisum sativum	DQ788719
Populus tremula x Populus tremuloides	EF125876

Populus trichocarpa	EF125877
Prunus domestica	AY860314
Psathyrostachys fragilis	AY271962
Robinia pseudoacacia	EF125871
Rosa hybrid	AY860315
Rosa rugosa	AY860316
Rubus idaeus	AY860317
Rumex acetosa	KC686838
Saccharum hybrid	KC686839
Saccharum officinarum	EF125872
Saruma henryi	EF125873
Secale cereale	AY359471
Silene latifolia	KC686837
Solanum lycopersicum	EU177767
Sorghum bicolor	AF538605, EF125875
Spartina alterniflora	AY603377
Sphaeropteris cooperi	AY860310
Triticum aestivum	AY271963
Vaccinium corymbosum	DQ673669
Zea mays	AF538618, AY271958, AY271959
Zingeria biebersteiniana	FJ975777, FJ975780
Zingeria kochii	FJ975778
Zingeria pisidica	FJ975779
Zingiber officinale	EF125874

Supplementary Table S2. Specific primers for Cassandra and 5S rRNA gene.

ID	Sequence (5'-3')	Location	Primer design source
**784**	CGAGTGAGGACAAAGTGCGCAG	161→182	LTR, Cassandra, *Hordeum vulgare*
**975**	ATCCCTAATAAGCCAGGCTATC	4←25	LTR, Cassandra, *Hordeum vulgare*
**977**	TTGTCCTCACTCATGCGCACC	153←173	LTR, Cassandra, *Hordeum vulgare*
**4170**	TGTAACCGCGAGGGTCGGCTCTGATACCA	269←297	PBS, Cassandra, *Hordeum vulgare*
**1032**	ACCGCGAGGGTCGGCTCTGATACCA	269←293	PBS, Cassandra, *Hordeum vulgare*
**981**	CATGACCGCACACCTGTCCG	308←327	Internal part, Cassandra, *Hordeum vulgare*
**982**	GACGAGGACGTCGGTTCCTCT	428→448	Internal part, Cassandra, *Hordeum vulgare*
**1801**	ACACCACCACTCAGAGGAACCGA	438←460	Internal part, Cassandra, *Hordeum vulgare*
**1802**	AGAGGAACCGACGTCCTCGTCG	427←448	Internal part, Cassandra, *Hordeum vulgare*
**4171**	CATGTAACCGCGAGGGTCGGCTCTG	275←299	Internal part, Cassandra, *Hordeum vulgare*
**3801**	GCTTGTGGTTGATCGACGAGG	435→455	Internal part, Cassandra, A*vena sativa*
**3802**	ACGCCCATGTAACCGCGAGG	302←321	Internal part, Cassandra, A*vena sativa*
**4172**	TGGGGTTGATCGACGAGGACGTCGATCT	438→465	Internal part, Cassandra, A*vena sativa*
**4173**	TGGGGTTGACCGACGAGGACGTCGGTCT	438→465	Internal part, Cassandra, A*vena sativa*
**4174**	GCTTGTGGTTGATCGACGAGGACG	435→458	Internal part, Cassandra, A*vena sativa*
**4175**	GGAACGTTCCTGTGGCCCGACGAGGACGTCG	410→440	Internal part, Cassandra, *Hordeum vulgare*
**2260**	ACACGCCCAAGCCAGATCGTCC	356←377	Internal part, Cassandra, Brachypodium
**2261**	ACTGACGAGGACGTCAGGTCC	794→814	Internal part, Cassandra, Brachypodium
**2070**	TCAGCGTGTTTTGTCCTCACTCG	306←328	Internal part, Cassandra, *Medicago truncatula*
**2071**	ACACGCTGAAAAGACTCGTGTTG	779→801	Internal part, Cassandra, *Medicago truncatula*
**921**	AGTGTGCCGACGAGGACGTCG	297←322	LTR-Internal part, Cassandra, *Malus*
**1611**	GGCTCTGATACCAAATTGTCACATCC	232←254	LTR, Cassandra, *Malus*
**2016**	AGGAGCTTGGGCGACCAAAGCGGAC	181→205	LTR, Cassandra, *Vaccinium*
**2495**	AGACTGTGTAGGGATGGGGCTA	139→160	LTR, Cassandra, *Garcinia mangostana*
**2496**	CATCCCTACACAGTCTCAGGAT	133←154	LTR, Cassandra, *Garcinia mangostana*
**631**	AGAACTCCAAAGTTAAGCGTGC	122→143	LTR, Cassandra, *Silene latifolia*
**629**	AGCACGCTTAACTTTGGAGTTC	123←144	LTR, Cassandra, *Silene latifolia*
**1118**	TAAGGTTTTGGGGTCTGGATGGCTAG	68→93	LTR, Cassandra, fern *Nephrolepis exaltata*
**1120**	TAAGGTGTTAGGAACCTCCGGTCTAGC	43←69	LTR, Cassandra, fern *Nephrolepis exaltata*
**622**	CTTCCCAGGAGGTCACCCATC	83←103	5S ribosomal RNA gene (121 bp)
**623**	TGATGGGTGACCTCCTGGGAAG	82→103	5S ribosomal RNA gene (121 bp)
**2721**	TGGGAAGTCCTCGTGTTGCA	97→116	5S ribosomal RNA gene (121 bp)
**2722**	AGTGCTGGTATGATCGCACC	3←22	5S ribosomal RNA gene (121 bp)

Supplementary Table S3. *Cassandra* tandem examples in EST and genome accessions.

GenBank accession	Sequence with Cassandra tandems (red is LTR, blue is internal part of *Cassandra*)
Saruma henryi (dt599514)	aaacagattgtacataccttttatgtgacgtcgtgcttgacttngagtaagttcaggatgggtgacctgaccgggaagtcttgttgaggggagccaaatgcgacaat<br>attattattattagagggtggggtgttacaaatggtatcagagccagtgcccagatgggggagcatgcacaagcctataaaagtcgccagttaagacgttgggtcc<br>taagagggggtgattgtagcgtcccatattacctgggtatggagatgggcttatatgtatacttacacccttaataataacaacgcattttaaagtcgtgatggcaa<br>tgatcccataagaactccgtagttaagcgtgctcgggtgagagcaatttcaggatgggtgacctgatcgggaagtcttgttgaggggagccaaaagcggataatatt<br>gttgttattagagggtggggtgttacaaatggcatcagagccaatacccagcctgagatgggggagcgtgcactagcccatgaaggtcgtcagcgaggacgctggg<br>tcctaagagggggtgattgtgacgtcccacatcgcctgggtatggagatgggactgtgcttgtatgtaaaatccacaccatttctaataacaacacgttttaaagat<br>gtgatggcaatgatcccataaaaactctgtagttaagcgtgtttggcttagagtaattttaggatgagtgacttgacggaaagtcttgttgaggggagccaaaagca<br>gacaatattgttgttattagagggtggggtgttacatttttgtaccaatgttgatatgtgaccccttcaaaagtcacc
Arabidopsis thaliana (al138645)	caactcaccgcattgaatctgatatttcctccctccactatttgccaaaaaagaaacaatttaaagaaacttatttataactcataaaacaacaaaatactcggata<br>ttgcttaaaacttaatgatttcaaatcttttctctctaatatcgtgcaatttttaaaagattttatccttaattagttgcaataatttttctttagccaccaagaa<br>agacctaatcgtctatatatacattttttgcaacactttttataaaataaatcctcaagttggattttgtttacaaggaatgtcattcgcattaattattatcaattaa<br>ttaatattcctaattactagtgtaacactccgagagaactccacagttaagtgtgcttgggtttgagtagtttcaggatgggtgaccttcggagaagtgattgtcgg<br>aaccatgtgagtgaggacaaagaatgggaaaagaaaatgtgataacttgaatagatggtaataatctttaaagcctcctggacgtgacgcactgactgttggacaga<br>ggtgggcccattggcccgagagaggacgtagggcccactgataggggcagtcgggacgttacaagtggtatctgagccaaacccataagagtgtagagtcaagaagg<br>ctcgcaccatcggggtatagccttggtgcgcaacgaggtcattgcgatctgttagtggggatgaattgtaacaccccgatttcaggaatattgtggtgcatgcggag<br>aggttttaacataatttatttggccacctatgtcaccaaaatgtacttatcttttcggtcaaaggtcttgagagaattccacagttaagcatgcttggactggaata<br>gtttcaagattggtgaccttccgagaagtgattgtcggaaccatgcgagtgagaacaaagcatgggaaaagctcatttggtgatttgtagggtcagtaacaagtaat<br>taaagcctcccaagcatggtgcaccgaccgtcggataggggtggatccattggccccagagattacgtggggcccacaaatagtggtggtcagagcattagaagtgg<br>tatcagagccgaacctaaatgagtgtggagtcaagaaggctcgcaccatcgggggttacgttcttactgcacaacgaacacgtaacaatctgttaatgcggtgaatt<br>gtaataccccggtttcaggaattggtttggccacctatgtcatcaaagtgcacttatcttttcggtcaaacatcctgagagaacgccacagttaagcatgcttgggc<br>tcgaatagttcaggatgggtgacctcccgggaagtgattgtcggaccaatgcgtgtgaggacaaagcatggagaatgatcatgtggtgatttgtagggatggtaaca<br>agtctttaaagcctctctcacgttacgcaccccaccattggacagaagtgagcccatgggcccaaaagagaacgtggggccaactggtaagggcggtcggggcgtta<br>caactagcatttcattatctaatatatctctatcggttattagaaaaatattaggtaattattaatattatactttattaaaggtaaatattatagttatttcaagt<br>ttattaaacatttagaatatttaacaaaaaaatgttttagaatatttataaatattaaaaaaaacatttgaaagaaaagttttaaattgatttgaattataatttct<br>taataaaagtattattaaaaacaacacgaaaaaataatgtttaatttcaccgaaccagattagatggtaggaagagtttagaatattttaagagaaaatttgggaaa<br>atgcctattttctaaattttttgtttgaaaaataacctaccccatttttgtattttaaaatatatttttttaaatagaaaaaaatataaaattaaaaaaatatccttat<br>taa
Lotus japonicas (ap006403)	ccgcaaggcccacccatatctcatctgttcttcgcggatgatgtacttctcttttgcaaagcctcctcaatacaagttcaattggtggcggacactttacaagcttt<br>ttgcactcattcaggtttgaagataaatatctcaaaatcaaaggctatttcttccaagggtgtgagatatgaaatcagagatgagatccggtccattgctcccatcc<br>cttttgtcaatgatcttgggcgttacttgggctttcccttgactggaggaaggatgtgacacccggggccgacgagggcggggagtgatcgccggtgcagtgaggca<br>cggacaaggagcggctcctggcaggcttctaggcggaagggcacatgactgaaccgatctcacacccgaacaagaggtattccgagattgtgtagggatggactata<br>cagttgaggagggcatatatgatttgattgtactacccataacaacaagttgcaacttcttttcgggagcccaactcataagaactccatggttaagtgtgctagcc<br>ttggatcaatattatgatgggtgacctcctgggaagttttcccgggaagcgcgcgagtgaggacaaagcgcgctgaaaagactcgtgttgttaccgtgaggttagtc<br>gtcacgtcaggatgttataattggtatcagagccgacctctcccagtacggtgtggttcggggacgaaccaagcggaagttggtgggcttgtgacacctggggccga<br>cgagggcggggagtgatcgccggtgcagtgaggcacagacaaggagcggctcctggcaggcttctaggcggagaggcacatgactgaaccgatctcacacccgaaca<br>agaggtattccgagattgtgtagggatggactatacagttgaggaaggcatatatgatttgattgtactacccataacaacaagttgcaacttcttttcgggagccc<br>aactcataagaactccatggttaagtgtgctagccttggatcaatattatgatgggtgacctcctgggaagttttcccgggaagcgcgcgagtgaggacaaagcgcg<br>ctgaaaagactcgtgttgttaccgtgaggttagtcgtcacgtcaggatgttacaattggtatcagagccacctctcccagtacggtgtggttcggggacgaaccaag<br>cggaagttggtgggcttgtgacacctgggcccgacgagggcggggagtgatcgccggtgcagtgaggcacggacaaggagcggctcctggcaggcttctaggcggag

	gggcacatgactgaaccgatctcacacccaaacaagaggtattccgagattgtgtagggatggactatacagttgaggagggcatatatgatttgattgtactaccc<br>ataacaacaagttgcaacttcttttcgggagcccaactcataagaactccatggttaagtgtgctagccttggagcaatattatgatgggtgacctcctgggaagtt<br>ttcctgggaagcgcgcgagtgaggacaaagcgcactgaaaagactcgtgttgttaccgtgaggtcagtcgtcacgtcaggatgttacaaaggacatctagaggctcc<br>tttaattattcattggagaatatctccaagaaacttgctacctggaaaactaatcttttgaattttgcagggaaaatttgtttaaccaaagcggtgattactgctat<br>gtcaacctatactatgcaggtgttctggttgcctaggtccatcattaacagcatgcaccaaatcatgagacgcgcctttgggcaaagggaaatggtaggcgaggttg<br>gcatttggtggggtggaatacgatcaccgaaagcaaacagcttggaggcctgggtgtgcgaaatttggcagaccacaatacagctctcctgggtaaggcactt
Malus	tgtaagatcccacatcgcccggaggaaaggaggtgatgtgctttatatgtacatactcacctccctatagaaagatgcgttttgggtggaagcccaagaacaaattt<br>gtgtgggcgtggtccaaagcggacaatatcttactatgtggactggatgtgacaattaggtatcagtgccactccccgaccggaagtgtgccgacgaggacgtcga<br>gcccctaaggggggtggattgtaagatcccacatcgcccggggagaggaggtgatgtgccttttacgtacagatattgtaagatcccacatcgctcgggggagagga<br>tgtgatatgtcttatatgtacatgtccacctccctatagagagatgcgttttgggtgaaagcccaagaacaaattcgtaagggcgtggcccaaagcggacaatatct<br>tactatgtggactgggatttgacaatttggtatcaaagccaatcccttgccggaagtgtgccgacgaggacgtcgagcccctaaggggggtggattgtaagatcccac<br>atcacccaggagataggaggtgatgtgtcttatatgtacatgcccacctccctatagagagacgcattttgggtggaaacctaagaacaaatccataagggcgtggc<br>ctaaagcggacactatcttactatgcggactgagatgtgaca
Malus (mdc000340.589:1..3145 phytozome)	acgaggagtcgggccctaagaggggtggattgtaacatcccacatcgcccatgggagtgatccttaaatgtgtattcccatccctacctagcacgaggcttttagg<br>agctcactggcttcgggttccatcggaactccgaagttaagtgagttcgcgcaagagcattcccatgatgggtgacccactgggaagttctcgtgtgagttcccaga<br>aacaaaaccgtgagggcgtggtcggggcccaaagcggacaatatcgtgctacggtggtggagcgggcccgggatgtggtgggggccaggccgggatgtgacaATTT<br>GGTATCAGAGCCTAACCCTGGCCGTGGTGTGCCGACGAGGACGTCGAGCCCCTAAGGGGGGtgaattgtaacatcccacatcgcctaggggagtgattcttaaatgt<br>gtattctcatccctacctagcacgaggccttttaggagctcactggcttcgggttccatcggaactccgaagttaagtgagttcgcgcgagagcattcccatgatgg<br>gtgacccactgggaakttctcgtgtggatttccagaaacaaaaccgtgagggcgtggtcagggctcaaagcggacaatatcgtgctacggtggtggagcggacccgg<br>gatgtggtaggggcccgggctgggatgtgacaATTTGGTATCAGAGCCTAACCCTGGCTGTGGTGTGCCGACGAGGACGTCGGGCCCCTAAGGGGGGtggattgtaa<br>catcccacatcgcccatgggagtgatccttaaatgtgtattcccatccctacctcgcacgaggccttttgggagctcactggcttcgggttccatcggaactccgaa<br>gttaagtgagttcgcgcaagagcattcccgtgatgggtgacccactgggaagttctcgtgtgagttcctagaaacaaaaccgtgagggcgtggtcggggcccaaagc<br>ggacaatatcgtgctacggtggtggagcggacccgagatgtggtgggggcccgggccgggatgtgacaATTTGGTATAAGAGCCTAACCTTGGCTGTGGTGTGCCGA<br>CGAGGACGTCGGGCCCCTAAgaggggtgrattgtaacatcccacatcacccatgggagtgatccttaaatgtgtattcccatccctacctagcacgaggccttttgg<br>gagatcactggcttcgggttccatcggaactccaaagttaagtgagttcgcgcgagagcattcccatgataggtgacccactgggaarttctcgtgtgagttcccag<br>aaacaaaaccgtgaggacgtggtarggacccaaagcggacaatattatgctacggtggtggagcgggcccgggatgtggtgggggccggggccgggatgtgacaATT<br>TGGTATCAGAGCCTAACCCTGGCCGTGGTGTGCCGACGAGGACGTCGGGCCCCTAAGGGGGGtggattgtaacatccccacatcgcctaggggagtgatccttaaat<br>gtgtattcccatccctacctagcacgaggcattttgggagctcactggcttcgggttccatcggaactccgaagttaagtgagttcgcgcgagagcattcccgtgat<br>gggtgacccactgggaagttctcgtgtgagttcccagaaacaaaaccgtgagggcgtggtcggggcccaaagcggacaatatcgtgctacggtgrtggagcggaccc<br>gggatgtggtgggggcccgggatgtgacaATTTGGTATCAGAGCCTAACCTTGGCTGTGGTGTGCCGACGAGGACGTCGGGCCCCTAAgaggggtggattgtaacat<br>cccacatcgcccatgrgagtgatccttaaatgtgtattcccatccctacctagcacgtggccttttgggagctcactggcttcgggttccatcgaaactccaaagtt<br>aagtgagttcgcgcgagagcattcccatgatgggtgacctactgggaagttctcgtgtgagttcccagaaataaaaccgtgaagacgtggtcgggacccaaagcgga<br>caatatcgtgctacggtggtggagcgggcccgggctgggatgtgacaATTTGGTATCAGAGCCTAACCCTGGCTGTGGTGTGCCGACGAGGACGTCGGGCCCCTAAG<br>GAGGGtggattgtaacatcccacatcgcccatgggagtgatccttaaatgtgtattcccatccctacctagcacgaggccttttgggagctcactggcttcgggttc<br>catcggaactccraagttaagtgagttcgcgcgagagcattcccatgatgggtgacccactgggaagttctcgtgtgagttcccagaaacaaaaccgtgagggcgtg<br>gtcggggcccaaagcgaacaatatcgtgctacggtggtggagcgggcccgggatgtggtgggggcctgggccgggatgtgacaATTTGGTATCAGAGCCTAACCATG<br>GCCGTGGTGTGCCGACGAGGACGTCGGGCCCCTAAGGGGGGtggattgtaacatcccacatcgcctaggggagtgatccttaaatgtgtattcccatccctacctag<br>cacgaggccttttgggagctcactggcttcgrgttccatcggaactccgaagttaagygagtwcgcgcgagagcawtcccatgatgggtgacccactgggaagttct<br>cgtgtgagttcccagaaacaaaaccgtgagggcgtaatcggggcccaaagcggacaatatcgtgctacggtggtggaatgagcccgggaagtggtccgccccgggcc<br>aggatgtgacaATTTGGTATCAGAGCCTAACCTTGGCCGCGTGTGTGCCGACGAGGACGTCGGGCCCCTAAGGGGGGtggattctgacatcccacatcgcccagggg<br>agtgatccttaaatgtatattcccatccctacctagcacgaggccttttgggagctcactggattcgagttccatcggaactccgaagttaagcgagtagcgcgcga<br>gagcaatcccatgatgggtgatccgctgggaagttctcgtgtgagttcccagaaacaaaaccgtgagggcgcagtcggggcccaaagcggacaatatagtgctacgg<br>tggtggaataggcccaggaagtgtccgccccgggcgggatgt

Prunus domesticus	tgtagaaaagagatacttttcatatatttgcagttcacgggtacaggctttgtatacaggcctaggaggtagctaaatggtttacaacggtagcgtagtaattata<br>tcaaacctaaatgctgtaattacaatacaataaagttatagttaattctatgatcattaagggatgatctgaatcctcaacatttcgatggatagggaagggtttg<br>ggaaagctagaagtaagactaacttgtagggtatgtagttggaaagcgtgctggaacttacaaaaaggtgtttgcttgcttttgaagcatgattctgaatctgatgc<br>gtagccattgccggaccacattgccaatccaccgctagacctaatttggattggttcagttaagggattaagctccaacctgtattgaactgatcccatgaaagacc<br>cgtgtcttggggaagcaagaacggtgagcaatcccacatcgctcggggagggaaaggggaagtgcttaaatgtgtgggctcacctattcctaacactaggccttttg<br>ggagctcattggcttcggaggggattggaactccgaagttaagcgagttgtgggctggagcaatcctatgatgggtgaccccctgggaagttgctcctgagctccca<br>gaaacaaaccgtgcgggcggctggtgagaatgtagccagcccaaagcggacaatatagtgctaggggagtcgggtcgtgacaatttggtatcagagccgacccgaca<br>ggaaatgtgccagcgaggacgctgggccccaagggggggtggattgtaagacccgtgtcttggggaagcaagaacggtgagcaatcccacatcgctcggggagggaaa<br>ggggaagtgtttaaatgtatgggttcgcctattcctagcactaggccttttgggagctcattggcttcggaggggattggaactccgaagttaagcgagttgtgggc<br>tggagcaatcccaggatgggtgaccccctgggaaattgctcctgagctcccagaaacaaaaccgtgcgggctggtgagaatgtagccaggcccaaagcagacaatat<br>agtgctaggggagtgccagcgaggacgctgggcctcaagggggggtggattgtaagacccgtgtcttggggaagcaagaactgtgagcaatcccacatcgctcgggga<br>gggaaaggggaagtgcttaaatgtatgggctcgcctattcctagcactaggccttttgggagctcattggcttcggaggggattggaactccgaagttaagcgagtt<br>gtgggctggagcaatcccaggatgggtgaccccctgggaagttgctcctgagctcgcagaaacaaaaccgtgcgggcggctggtgagaatgtagccaggcccaaagc<br>ggacaatatagtgctaggggagtcgggtcgtgaca
Fragaria	acgaggccttttgggagctcactggcttcggaggggatgggtactccgaggttaagcatgttgatgctaaagcaatctcaggatgggtgacctactgggaaggtgct<br>cctgagctgccggaaacaaaaccgtgagggccggtgggcccaaagcggagaatatcgtgttacggcggaatgagtcctgggatgtgacaaatggtatcagagccact<br>ctgtcgtgtggtgcgagtgtgccgacgagggcgtcggactcccaagggggggtggattgtaatatcccacatcggccaaacggagagaggttatgtgctgtatatgta<br>catgcccacctctaatctaacacgaggccttttggtggctcagcggcttcggaggggatgggtactccgaggttaagtatgttgatgctagagcaatcccaggatgg<br>gtgacctactgggaagctgctcctgagctgccggaaacaaaaccgtgagggccggcgggcccaaagcggacaatatcgtgttacggcgaaatgggtcctgggatgtg<br>acaatggtataagatccactctgccgtgtggtgcgagtgtgccgacgagggcgtcggactcccaagggaggggggatggattgtaatatcccacatcggccaaacgaa<br>gaggggttatgtgccttatatgtacatgcccacctcca

Supplementary Table S4. Relative *Cassandra* copy numbers per 1C in *H.spontaneum* Evolution Canyon samples and for *H.vulgare* cultivars measured using dot blot hybridization and qPCR.

H.spontaneum **Evolution Canyon lines**	***Cassandra*** **element copy number**		***Cassandra*** **tandem[2] copy number by qPCR**
	**by dot blot**	**by qPCR [1]**	
1-SH		3061	632
2-SH	4333	2454	543
3-SH		1278	272
4-SH		1601	400
5-SH	4237	3023	581
6-SH		2644	404
7-SH		1316	244
8-SH		2075	351
9-SH		2094	351
Avg. ± SD	4285 ±66.525 (±1.55%)	2172 ±418 (±19.26%)	420 ±84 (±20.03%)
11-SM		2170	369
12-SM		2340	400
13-SM		2720	464
14-SM	5437	2985	548
15-SM		3536	680
16-SM		2606	474
17-SM		3004	528
18-SM		3422	687
19-SM		2511	438
20-SM		2625	474
Avg. ± SD	5437	2792 ±261 (±9.34%)	506 ±63 (±12.47%)
21-SL		1980	313
22-SL	5032	3365	680
23-SL		2758	483
24-SL		3536	751
25-SL		3023	548
26-SL		3555	626
27-SL	5859	1449	237
28-SL		3004	518
29-SL		2853	412
30-SL		2720	460
Avg. ± SD	5446 ±573 (±10.52%)	2824 ±393 (±13.92%)	503 ±94 (±18.60%)
31-NL		2891	493
32-NL		2910	488
33-NL		3118	533
34-NL	3896	3896	575
35-NL		3403	400
36-NL		2340	272
37-NL		3592	433

39-NL		2872	328
40-NL		2720	285
Avg. ± SD	3896	3082 ±294 (±9.53%)	423 ±67 (±15.94%)
42-NM	4389	3232	469
43-NM		4674	1150
44-NM		3839	493
45-NM		2815	304
46-NM		2208	252
47-NM	5123	3517	416
48-NM		3384	442
49-NM		3668	513
Avg. ± SD	4756 ±509 (±10.69%)	3417 ±471 (±13.77%)	505 ±179 (±35.46%)
51-NH		1980	222
52-NH		3479	451
53-NH	5081	5736	848
54-NH		2853	292
55-NH		2056	226
57-NH		3365	508
58-NH		2587	261
59-NH	6040	1752	200
60-NH		3877	381
61-NH		3137	408
Avg. ± SD	5561 ±665 (±11.95%)	3082 ±686 (±22.26%)	380 ±115 (±30.29%)
H.vulgare, winter barley, 424 (Tu Da Mai 1, China)	6732		
H.vulgare, winter barley, 431 (Han 85-222, China)	7364		
H.vulgare, winter barley, 833 (Casbon, USA)	5930		
H.vulgare, winter barley, 938 (Tennessee Winter, USA)	6761		
H.vulgare , Kymppi, (callus)	5550		

[1] PCR fragment with primers: 784 and 977

[2] PCR fragment with primers: 981 and 982
